# “HE EXPLAINED IT TO ME AND I ALSO DID IT MYSELF”: HOW OLDER ADULTS GET HELP WITH DIGITAL TECHNOLOGY

**DOI:** 10.1093/geroni/igz038.3179

**Published:** 2019-11-08

**Authors:** Amanda E Hunsaker, Minh Hao Nguyen, Jaelle Fuchs, Teodora Djukaric, Larissa Hugentobler, Eszter Hargittai

**Affiliations:** University of Zurich, Zurich, Switzerland

## Abstract

Older adults comprise a highly heterogeneous group that engages with digital media in varying ways, therefore a large variation in technology support needs is likely. This study examines the nature of support for using digital media among older adults. We conducted in-depth qualitative interviews with older adults (age 59+) in Hungary, the Netherlands, and Switzerland (N=58) in 2019 exploring: (1) whether and how older adults receive support in using digital media; and (2) older adults’ perceptions of whether the support they receive meets their needs. We began with open coding, then conducted consensus meetings to identify themes and coding schemes, and wrote memos to share findings and ensure reliability across coders. We find that older adults voice a highly varying range of need for technical support as well as varying instances of both receiving and not receiving technical help. Participants report receiving help from different informal (e.g. spouses) and formal (e.g. computer classes) sources. However, support may not be immediate, posing challenges for older adults who depend on the availability of their support sources. Importantly, we also find that there are older adults who are quite self-sufficient in the ways they use digital technology. For older adults needing support, greater access to community-based support may help those without satisfactory options in their own social circle. Given our findings that older adults can have great ease with solving technology-related problems, peer-driven support networks where older adults can offer support to others may be an effective approach to providing digital technology guidance.

